# Social Trust Profile Transitions and Health-Seeking Behavior: A Latent Transition Analysis of Three-Wave Panel Data from China

**DOI:** 10.3390/bs16071189

**Published:** 2026-07-14

**Authors:** Yuhan Wu, Shihan Xu, Lu Li

**Affiliations:** 1 School of Health Management, Guangzhou Medical University, Guangzhou 511436, China; wuyuhan@stu.gzhmu.edu.cn (Y.W.); xushihan@stu.gzhmu.edu.cn (S.X.); 2 Guangdong-Hong Kong-Macao Greater Bay Area Medical and Health Industry High Quality Development Rule of Law Guarantee Research Center, Guangzhou 511436, China; 3 Philosophy and Social Sciences Key Laboratory, Guangdong Higher Education Institutes for Health, Guangzhou 511436, China

**Keywords:** social trust, latent transition analysis, health-seeking behavior, longitudinal panel data

## Abstract

Social trust is increasingly recognized as a psychosocial determinant of health service utilization, yet most studies treat trust as a unidimensional, static variable. This study examines how multidimensional trust configurations evolve over time and whether distinct profiles are associated with subsequent health-seeking behavior. Data were drawn from three waves (2018, 2020, 2022) of the China Family Panel Studies (N = 12,216). Latent profile analysis identified trust configurations based on four indicators (trust in neighbors, strangers, government officials, and doctors), and a three-wave latent transition analysis with partial measurement invariance modeled profile stability and change. Time-lagged logistic regressions and stayer–mover analyses examined associations with care-seeking. Three profiles emerged: Low Trust (31%), Higher Generalized Trust (36–42%), and Selective Institutional Trust (27–33%). All profiles showed moderate-to-high stability. Selective Institutional Trust was consistently associated with higher odds of care-seeking at the subsequent wave (adjusted OR = 1.30–1.35, *p* < 0.01), an association strongest among those who maintained this profile over time (OR = 1.47–1.48, *p* < 0.001). Higher Generalized Trust showed no significant association, and no profile was associated with facility-level choice. These findings indicate that the behavioral relevance of social trust for health-seeking is profile-specific rather than monotonic, underscoring the value of person-centered longitudinal approaches.

## 1. Introduction

Health-seeking behavior (HSB) refers to actions taken by individuals after perceiving a health problem to obtain information, diagnosis, treatment, or symptom relief (Zhou et al., 2021). HSB has important implications for disease recognition, timely treatment, chronic disease management, and the efficiency of health service utilization (Lau et al., 2020; Zhou et al., 2021). Although care-seeking is shaped by service availability, affordability, and institutional incentives, individuals facing similar structural conditions may still differ in whether, when, and where they seek care. This suggests that psychosocial factors are also important for explaining variation in health-seeking behavior (Atherton et al., 2024; Lau et al., 2020).

Among these, social trust has received increasing attention (Paquin et al., 2025). According to the Andersen Behavioral Model, health service utilization is shaped by predisposing characteristics, enabling resources, and need factors (Andersen, 1995). Within this framework, social trust can be understood as a psychosocial predisposition: it may be related to how individuals judge the credibility of medical information, evaluate uncertainty in the health system, and decide whether to use formal medical services (Arakelyan et al., 2021; Connolly et al., 2023). Social trust is also a core component of cognitive social capital. It may be associated with where individuals obtain health information and whether they regard public institutions and professional services as legitimate and reliable (Gilson, 2003; Putnam, 2000).

However, social trust is not a unidimensional construct. Individuals may hold different levels of trust toward neighbors, strangers, government, and doctors, and these dimensions do not necessarily move together (Hall et al., 2001). These four targets were selected because they capture the interpersonal–institutional spectrum of trust most relevant to health-seeking behavior. Trust in neighbors and strangers taps into interpersonal trust, whereas trust in government officials and doctors taps into institutional trust (Arakelyan et al., 2021; Gilson, 2003). Within the social capital framework, these indicators operationalize cognitive social capital, defined as perceptions of trust, reciprocity, and belonging in one’s social environment (Harpham et al., 2002). Within the Andersen Behavioral Model, such trust attitudes function as psychosocial predisposing factors that influence how individuals evaluate health information, perceive system uncertainty, and decide whether to use formal services (Andersen, 1995). This multidimensional operationalization is particularly relevant in China, where informal networks and formal institutions may exert competing influences on care-seeking (Lau et al., 2020; Zhou et al., 2021). For example, some individuals may trust the government and doctors while remaining cautious toward strangers, whereas others may show low trust across most objects. Treating trust as a single score or simple average may therefore obscure meaningful multidimensional configurations and may explain why similar overall levels of trust correspond to different health-seeking behaviors.

Three gaps remain in the literature. First, many studies model trust as a single variable, with limited attention to how different trust objects combine within individuals. Second, most evidence is cross-sectional, making it difficult to examine whether trust states are stable, whether they change over time, and how such changes occur. Third, limited evidence links social trust profiles and their longitudinal transitions to subsequent health-seeking behavior, particularly using large-scale longitudinal panel data from China. Latent profile analysis (LPA) and latent transition analysis (LTA) provide tools for addressing these gaps: LPA identifies heterogeneous trust configurations, while LTA characterizes movement between trust states over time (Nylund-Gibson et al., 2023; Paquin et al., 2025).

China provides an important setting for this question. The tiered healthcare delivery system and primary care gatekeeping policies aim to guide residents toward more structured care pathways, yet the decision to seek care and the choice of facility level may reflect different mechanisms (Shen & Zhang, 2016; Zhou et al., 2021). It is therefore useful to distinguish between entering the formal health system and choosing a primary care facility after deciding to seek care.

Using three waves of CFPS data from 2018 (T1), 2020 (T2), and 2022 (T3), this study identified multidimensional social trust profiles among Chinese adults, examined their longitudinal transitions, and tested their associations with subsequent illness-related care-seeking behavior. Specifically, we examined: (1) whether interpretable multidimensional trust profiles exist; (2) how these profiles remain stable or change over time; (3) whether trust profiles and transition pathways are associated with later health-seeking behavior; and (4) whether the profile structure remains robust after excluding doctor trust. This study extends prior research by applying a person-centered longitudinal approach to multidimensional trust and care-seeking, using time-lagged models to strengthen temporal sequencing relative to cross-sectional designs, and assessing measurement invariance to support cross-wave comparability of profiles.

## 2. Materials and Methods

### 2.1. Data and Sample

Data were drawn from the China Family Panel Studies (CFPS), a nationally representative longitudinal household survey conducted biennially since 2010 (Xie et al., 2014). We used three consecutive waves (2018, 2020, and 2022) to construct a balanced panel of adults aged 18 years or older who were successfully followed across waves and had valid responses to all four trust items at each wave.

**Figure 1:**
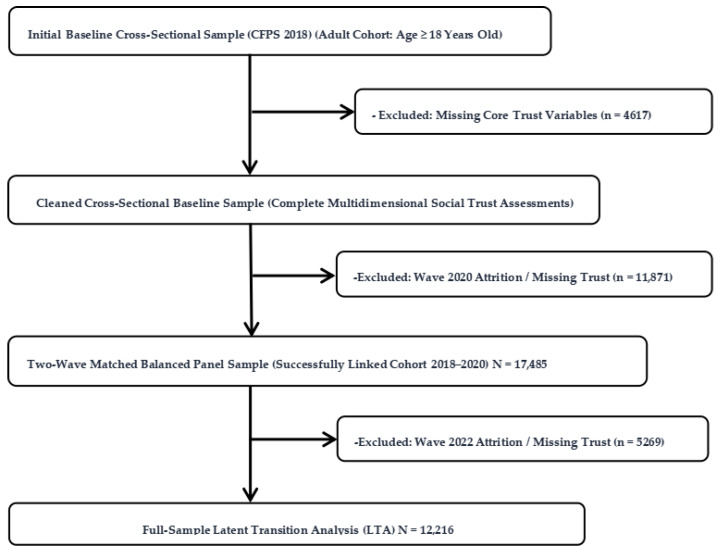
Flow chart of sample selection.

Sample construction proceeded as follows. The 2018 baseline adult sample included 33,973 respondents. After excluding 4617 respondents with missing or invalid responses to the four trust items, 29,356 remained. Cross-wave matching retained 17,485 respondents in 2020, and further matching with the 2022 wave produced a final balanced panel of 12,216 respondents (50.5% female; mean age = 44.9 years, SD = 14.9). Attrition from 29,356 to 12,216 may introduce selective bias, which is addressed in the Limitations section. The detailed flowchart of the sample selection process is presented in Figure 1.

According to the CFPS skip pattern, care-seeking questions were administered only to respondents who reported physical discomfort or illness in the preceding two weeks. Regression analyses of health-seeking behavior were therefore restricted to respondents reporting recent illness at the relevant wave (approximately 3200–3700 per wave), focusing on individuals with an active health need. Consequently, the regression findings should be interpreted as applying to adults experiencing recent illness rather than to the general adult population.

Of the 33,973 baseline adults, 4617 (13.6%) had missing or invalid responses on at least one trust item and were excluded. To evaluate attrition-related selection, we compared retained and lost respondents on all available baseline characteristics (standardized mean differences [SMD] and significance tests) and estimated a logistic retention model from which stabilized inverse-probability-of-retention weights were constructed (truncated at the 1st and 99th percentiles; Seaman & White, 2013). Retention was related mainly to observed baseline age, consistent with a missing-at-random mechanism conditional on observed covariates; weighted sensitivity results are reported in Section 3.6 and Appendix B.

### 2.2. Measures

#### 2.2.1. Trust Indicators

Social trust was measured using four items assessing trust in neighbors, strangers, government officials, and doctors. Each item was rated on an 11-point scale ranging from 0 (no trust at all) to 10 (complete trust). The same items were available across the three waves and were treated as continuous indicators in the LPA and LTA models. Invalid responses, including refusal, “don’t know,” and “not applicable,” were coded as missing.

#### 2.2.2. Health-Seeking Outcomes

The primary outcome was whether the respondent sought medical care after recent physical discomfort or illness. Respondents who reported visiting a hospital or clinic were coded as 1, and those who did not seek care were coded as 0. A secondary exploratory outcome, constructed among care-seekers only, indicated whether the visit was to a primary care facility, defined as a community health center, township hospital, or village clinic, versus a higher-level institution.

#### 2.2.3. Covariates

Covariates were measured at baseline in 2018 and selected following the Andersen Behavioral Model (Andersen, 1995). In the profile–outcome regressions, covariates were entered hierarchically: Model 1 included age and sex; Model 2 added self-rated health, chronic disease status, and urban/rural residence; Model 3 further added years of education, marital status, and health insurance coverage. In the transition-predictor models, covariates included satisfaction with access to care, satisfaction with care quality, hospitalization in the past year, and baseline illness status.

### 2.3. Statistical Analysis

#### 2.3.1. Latent Profile Analysis

Within each wave, two- to five-profile models were estimated using latent profile analysis (LPA). Model selection was based on AIC, BIC, sample-size adjusted BIC (aBIC), entropy, the bootstrap likelihood ratio test (BLRT), minimum profile size, and substantive interpretability (Akaike, 1974; Atherton et al., 2024; Muthén & Muthén, 2000; Petersen et al., 2022).

#### 2.3.2. Latent Transition Analysis and Measurement Invariance

Based on the LPA results, a three-profile latent transition analysis (LTA) was fitted across the three waves using robust maximum likelihood estimation (MLR). Multiple random starts were used, and replication of the best log-likelihood was required to reduce the risk of local solutions. To assess cross-wave comparability of the profiles, we compared three measurement specifications: a freely estimated model, in which all indicator means were freely estimated across waves; a full longitudinal invariance model, in which all indicator means were constrained equal across waves within corresponding profiles; and a partial invariance model, in which neighbor and stranger trust were constrained equal across waves while government and doctor trust were freely estimated. The final model was selected based on relative fit, classification quality, profile interpretability, and stability of substantive results. Subsequent profile membership, transition pathways, and regression analyses were based on modal posterior profile assignments from the selected LTA model.

#### 2.3.3. Profile–Outcome Associations

Time-lagged binary logistic regressions estimated associations between trust profile membership at wave t and health-seeking behavior at wave t + 1 (2018 → 2020 and 2020 → 2022), with the Low-Trust profile as the reference category. Odds ratios (ORs) and 95% confidence intervals are reported. Regression models used complete cases for covariates; therefore, analytic sample sizes varied slightly across model specifications. In addition, a stayer–mover framework (Wells et al., 2018) was used to compare care-seeking behavior across longitudinal trajectory groups, and logistic regression models identified predictors of profile transitions.

Robustness and sensitivity analyses. Five additional checks were performed. First, all regression, stayer–mover, and transition-predictor analyses were re-estimated under the freely estimated measurement model to assess sensitivity to measurement invariance assumptions. Second, the LTA was re-estimated after excluding the doctor-trust indicator to test whether the profile structure depended on this health-domain-specific item. Third, the fully adjusted care-seeking models were re-estimated with attrition-based inverse-probability weights. Fourth, to address classification uncertainty in modal assignment, we report average posterior probabilities by wave and profile, and re-estimated the outcome models (a) weighting observations by their posterior profile probabilities (proportional assignment) and (b) using pseudo-class multiple imputation with 200 random draws combined by Rubin’s rules (Bandeen-Roche et al., 1997; Wang et al., 2005; Vermunt, 2010; Asparouhov & Muthén, 2014). Automatic R3STEP/BCH routines were not directly implemented because the model links profile membership across three waves and includes a skip-pattern distal outcome. Fifth, to address contextual confounding, province identifiers were merged from the original CFPS files and the fully adjusted models were re-estimated with province fixed effects, absorbing all time-invariant province-level differences in healthcare capacity, economic development, and expenditure. In addition, covariate-adjusted average predicted probabilities of care-seeking by profile were computed from the fully adjusted models. Results of all checks are reported in Section 3.6 and Appendix B.

LPA and LTA were conducted in Mplus 8.3. Data management and regression analyses were performed in Stata 18 and R 4.0.5.

## 3. Results

### 3.1. Sample Characteristics

The final analytic sample comprised 12,216 respondents. At baseline, 50.5% were female, with a mean age of 44.9 years (SD = 14.9). Urban residents accounted for 52.3%, mean self-rated health was 3.03 (SD = 1.18), and 15.5% reported a chronic condition. Mean years of education was 8.55 (SD = 4.76), 82.5% were married, 92.2% had health insurance, and 11.3% had been hospitalized in the past 12 months (Table 1).

Table 2 presents trust indicator means across waves. Neighbor trust remained stable (6.74, 6.73, 6.65), stranger trust increased gradually (2.33, 2.39, 2.65), government trust rose from 5.01 in 2018 to 5.69 in 2020 and declined to 5.52 in 2022, and doctor trust followed a similar pattern (6.67, 7.08, 6.91). The two-week illness rate was 30.1% in 2018, 26.4% in 2020, and 27.2% in 2022. Among those reporting illness, 64.2% sought medical care in 2020 and 64.0% in 2022.

### 3.2. Profile Enumeration and Model Selection

Within each wave, two- to five-profile LPA models were estimated (Table 3A). All BLRTs were significant, and information criteria decreased as additional profiles were added. The three-profile solution was retained at all waves as the most interpretable and statistically supported solution. Two-profile models showed low entropy (0.63–0.66), while models with four or more profiles produced classes smaller than 5% in at least one wave, with the smallest class comprising 2.4% of the sample at T3 in the four-profile solution and 2.6–2.8% at T1–T2 in the five-profile solution. Such sparse classes tend to have limited generalizability and are less likely to replicate in independent samples, and additional profiles offered limited substantive differentiation from existing profiles, limiting interpretability. The 2018 three-profile solution had an entropy of 0.719, indicating moderate classification precision in that wave and a greater possibility of class-assignment uncertainty compared with later waves. We therefore interpret the T1–T2 results with caution, while noting that the core associations were replicated in the T2–T3 interval where classification quality was higher.

In the combined three-wave LTA, the three-profile model outperformed the two-profile model on all fit indices (ΔAIC = 9268; ΔBIC = 9112; entropy 0.793 vs. 0.763; Table 3B).

To support cross-wave comparability, three measurement specifications were compared (Table 3C). The freely estimated model (62 parameters, BIC = 627,878, entropy = 0.793) fit best but was least parsimonious. The full invariance model (38 parameters, BIC = 628,380, ΔBIC = +502, entropy = 0.790) fit substantially worse and was rejected. The partial invariance model constrained neighbor and stranger trust to be equal across waves while allowing government and doctor trust to vary freely (50 parameters, BIC = 627,889, ΔBIC = +11, entropy = 0.792), achieving comparable fit with fewer parameters. This model was adopted for all subsequent analyses, as it supported cross-wave comparability while accommodating time-specific variation in institutional and doctor trust.

### 3.3. Profile Characteristics and Composition

**Figure 2:**
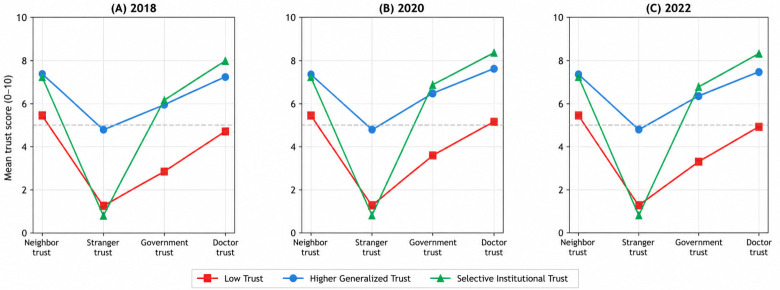
Trust profile indicator means across three waves (partial invariance model).

Three profiles showed consistent patterns across waves (Figure 2). To provide precise quantification for these trust configurations, the estimated indicator means across the three waves under the partial measurement invariance model are detailed in Table A1 in Appendix A.1. The Low-Trust profile scored lowest on all indicators. The Higher Generalized Trust profile scored moderately high across all dimensions, including stranger trust. The Selective Institutional Trust profile combined very-low stranger trust (approximately 0.8) with high government and doctor trust and high neighbor trust (>7), producing a distinctive V-shaped pattern.

Profile composition shifted over time (Table 3D): the Low-Trust profile remained relatively stable (31.0%, 31.4%, 30.3%); the Higher Generalized Trust profile expanded (35.5%, 37.6%, 42.4%); and the Selective Institutional Trust profile contracted (33.4%, 31.0%, 27.3%).

### 3.4. Profile Transitions

Table 4 presents transition probability matrices for both intervals. All profiles showed moderate-to-high stability. The Low-Trust profile had the highest retention (78.2% and 76.6%), followed by Higher Generalized Trust (73.3% rising to 80.1%) and Selective Institutional Trust (69.9% and 69.7%).

Off-diagonal probabilities indicated that Low-Trust individuals were most likely to transition to Higher Generalized Trust (12.1%, 15.8%) rather than directly to Selective Institutional Trust. Transitions from Selective Institutional Trust favored Higher Generalized Trust (21.0%, 22.2%) over Low Trust (9.2%, 8.1%).

### 3.5. Trust Profiles, Transition Pathways, and Health-Seeking Behavior

Profile–outcome associations. Time-lagged logistic regressions with hierarchical covariate adjustment showed that Selective Institutional Trust was associated with higher odds of seeking care at the subsequent wave, relative to Low Trust (Table 5A). For the 2018 → 2020 interval, Model 1 (age, sex) yielded OR = 1.35 (95% CI [1.13, 1.62], *p* = 0.001); Model 2 (+self-rated health, chronic disease, urban residence) yielded OR = 1.30 (*p* = 0.005); and Model 3 (+education, marital status, insurance) yielded OR = 1.30 (*p* = 0.006). For the 2020 → 2022 interval, Model 1 yielded OR = 1.43 (95% CI [1.19, 1.71], *p* < 0.001); Model 2 yielded OR = 1.36 (*p* = 0.001); and Model 3 yielded OR = 1.35 (*p* = 0.002). Higher Generalized Trust was not significantly associated with care-seeking in any model (OR range 1.04–1.08, all *p* > 0.27).

Among care-seekers, neither profile was significantly associated with choosing primary care over higher-level facilities in either interval (Table 5B; all *p* > 0.14).

To aid interpretation of effect magnitude, covariate-adjusted average predicted probabilities of seeking care when ill were 62.0% (95% CI 59.3–64.7%) for Low Trust versus 67.6% (95% CI 64.8–70.4%) for Selective Institutional Trust in the 2018 → 2020 interval. The corresponding probabilities were 61.6% versus 68.0% in the 2020 → 2022 interval, representing absolute differences of 5.6 and 6.5 percentage points, respectively. Higher Generalized Trust fell in between (64.2% and 63.3%; Appendix B Table A7).

Stayer–mover analysis. Compared with the Stable Low-Trust pathway, the Stable Selective Institutional Trust pathway was associated with significantly higher care-seeking in both intervals (2018 → 2020: N = 790, OR = 1.47, *p* < 0.001; 2020 → 2022: N = 731, OR = 1.48, *p* < 0.001; Table 5C). The Stable Higher Generalized Trust pathway did not differ from Stable Low Trust in either interval. The Selective-to-Low transition pathway showed elevated care-seeking in the first interval (N = 85, OR = 1.86, *p* = 0.020) but not the second (N = 117, OR = 0.98, *p* = 0.92); given the small samples and inconsistent results, this finding is not interpreted further.

Predictors of profile transitions. Among Low-Trust individuals (N = 3788; transition rate 8.4%), satisfaction with access to care was associated with transition to Selective Institutional Trust (OR = 1.41, *p* < 0.001), while past-year hospitalization was negatively associated (OR = 0.60, *p* = 0.020; Table 5D). Among Higher Generalized Trust individuals (N = 4343; transition rate 12.0%), satisfaction with access to care was protective against transition to Low Trust (OR = 0.81, *p* = 0.004), and older age was associated with higher transition probability (OR = 1.01, *p* < 0.001). No predictor reached significance for the Selective-to-Low transition (N = 4085; transition rate 7.5%).

### 3.6. Sensitivity Analyses

To verify the robustness of the profile structure, re-estimating the LTA after excluding the doctor-trust indicator replicated the three-profile structure (entropy = 0.799; retention probabilities 71–82%), with broadly similar profile composition (largest difference approximately six percentage points, for the Selective Institutional Trust profile) and substantive interpretations, indicating that the Selective Institutional Trust profile was not driven by doctor trust alone (detailed model parameters are presented in Table A3 in Appendix A.3). To examine the sensitivity to model constraints, re-estimating all regression, stayer–mover, and transition-predictor models under the freely estimated measurement specification produced consistent results in direction and significance, with key associations showing only marginal changes in magnitude (e.g., the adjusted OR for Selective Institutional Trust predicting care-seeking in the 2018 → 2020 interval shifted from 1.30 [95% CI 1.08, 1.56] to 1.34 [95% CI 1.11, 1.61]), indicating that the main findings were robust to measurement specification (detailed estimates are provided in Table A2 in Appendix A.2).

Additional analyses addressed attrition and classification uncertainty (Appendix B). Respondents lost to follow-up were older than retained respondents (49.1 vs. 44.9 years, SMD = 0.26), but differed negligibly on all four trust indicators (SMDs 0.00–0.06; Table A4). Re-estimating the fully adjusted models with stabilized inverse-probability-of-retention weights did not materially alter the results (Selective Institutional Trust: OR = 1.29, *p* = 0.007 for 2018 → 2020; OR = 1.33, *p* = 0.003 for 2020 → 2022; Table A5). Average posterior probabilities for the assigned profile ranged from 0.865 to 0.929 across all wave-by-profile combinations (Table A6). Models weighting observations by posterior profile probabilities and pseudo-class multiple-imputation models (200 draws) reproduced the main findings (Selective Institutional Trust OR = 1.31 and 1.37 in the two intervals under both procedures; Table A8). Finally, adding province fixed effects to the fully adjusted models yielded OR = 1.25 (95% CI 1.04–1.51, *p* = 0.020) and OR = 1.34 (95% CI 1.11–1.62, *p* = 0.003), indicating that time-invariant province-level factors do not account for the main association (Table A9).

## 4. Discussion

### 4.1. Main Findings

The most consistent finding of this study is that the Selective Institutional Trust profile, characterized by high trust in government and doctors, high neighbor trust, and very low stranger trust, was significantly associated with higher odds of seeking medical care following illness. This association was replicated across two independent two-year intervals, remained robust after hierarchical adjustment for demographics, health needs, and socioeconomic factors, and was strongest among individuals who maintained this profile over time. By contrast, the Higher Generalized Trust profile showed no significant association with care-seeking in any model. Among those who did seek care, no trust profile was associated with choice of primary versus higher-level facilities.

These findings indicate that the behavioral relevance of trust is profile-specific rather than monotonic (Ye et al., 2024). Higher Generalized Trust does not necessarily translate into greater health service use. Rather, high institutional trust combined with low stranger trust is the configuration most consistently linked to subsequent engagement with the formal healthcare system.

Although the odds ratios of 1.30–1.47 for the Selective Institutional Trust profile are modest in magnitude, their replication across two independent two-year intervals and the stronger association observed among stable profile members (OR = 1.47–1.48) suggest a non-negligible behavioral relevance in the context of health-seeking, where decisions are shaped by multiple structural and need-based factors.

This pattern is consistent with the Andersen Behavioral Model (Andersen, 1995), in which trust functions as a psychosocial predisposing factor that may be related to how individuals evaluate the legitimacy of formal healthcare institutions and whether they act on health needs. Gilson (2003) theorized that institutional trust reduces perceived uncertainty and transaction costs in health system interactions; the present longitudinal evidence is consistent with this proposition. From a social capital perspective (Putnam, 2000; Szreter & Woolcock, 2004), different forms of trust may channel individuals toward different information sources. Selective Institutional Trust individuals, who distrust generalized strangers but trust formal institutions, may preferentially rely on authoritative medical information rather than informal networks, thereby being associated with a greater likelihood of formal care-seeking.

The finding that the profile structure was not solely dependent on doctor trust, as shown by the sensitivity analysis excluding this indicator, suggests that the observed association may reflect a broader institutional-trust configuration (Meyer et al., 2024) rather than physician trust alone (Calnan & Sanford, 2004). Gopichandran and Sakthivel (2021) similarly found that institutional trust and interpersonal trust in doctors are separable dimensions (Krastev et al., 2023), with institutional trust playing a foundational role in care-seeking decisions. Ozawa and Sripad (2013), in their systematic review of trust and healthcare in low- and middle-income countries, also emphasized that trust shapes willingness to engage with health systems. These conclusions are consistent with the present finding that Selective Institutional Trust was associated with care-seeking, whereas it was not associated with facility-level choice.

The null finding for primary care facility choice suggests that trust in the health system does not directly promote primary care utilization. Our results suggest that facility-level choice is likely governed by structural factors, such as service capacity, insurance reimbursement differentials, and referral mechanisms, rather than by trust attitudes alone.

### 4.2. Trust Dynamics and Service Experience

Trust profiles showed moderate-to-high stability across adjacent waves, yet meaningful transitions did occur. Low-Trust individuals were most likely to transition to Higher Generalized Trust rather than directly to Selective Institutional Trust, which may suggest a gradual broadening of trust rather than an immediate shift toward institutional trust (Sønderskov & Dinesen, 2016). Selective Institutional Trust individuals who transitioned moved predominantly toward Higher Generalized Trust rather than Low Trust, indicating that this profile was not an absorbing state.

Government and doctor trust showed notable fluctuation around 2020 (Skirbekk et al., 2023), consistent with the COVID-19 pandemic context. Research on trust during public health crises has documented heterogeneous shifts in institutional trust across population subgroups (Algan et al., 2021; You et al., 2024). The partial invariance model adopted in this study accommodated such time-specific variation in government and doctor trust while preserving cross-wave comparability for neighbor and stranger trust, making it well suited to this data structure.

A closer examination of the profile indicator means revealed a similar temporal pattern across all three trust profiles. Government trust and doctor trust generally increased from 2018 to 2020 and then declined modestly between 2020 and 2022, although remaining above their 2018 levels. This pattern may reflect the unique social and institutional context of the COVID-19 pandemic rather than solely long-term individual trust evolution. During major public health crises, institutional trust may become more responsive to government performance, healthcare delivery, and public health communication than under routine conditions. The subsequent decline observed in 2022 may therefore indicate a partial normalization of institutional trust as the acute phase of the crisis subsided. Previous studies have likewise suggested that increases in institutional trust during public health emergencies are not always permanent and may diminish as perceived threat decreases (You et al., 2024). Accordingly, some of the trust transitions observed between 2020 and 2022 may reflect period-specific influences associated with the pandemic context, in addition to longer-term individual-level changes in trust.

Satisfaction with access to care emerged as a significant predictor of trust transitions (Akafu et al., 2023). Higher satisfaction was associated with a higher likelihood of transition from Low Trust to Selective Institutional Trust and was associated with a lower likelihood of transition from Higher Generalized Trust to Low Trust. This suggests that trust change may be partly related to concrete service experiences rather than to dispositional factors alone. Improving the convenience and accessibility of formal health services may be associated with trust development among Low-Trust populations (Kruk et al., 2018).

### 4.3. Implications for Health Service Use

Two implications follow from these findings. First, improving care-seeking requires attention not only to financial barriers (HaGani et al., 2023) but also to the psychosocial trust foundation (Gao et al., 2024) underlying health system engagement. Expanding insurance coverage and service supply may not, by themselves, be sufficient to increase willingness to engage with the health system. Second, trust-building efforts should distinguish among objects of trust. Generalized High Trust was not reliably associated with care-seeking, whereas trust in formal institutional actors, including government and medical professionals, appeared more directly relevant to the decision to seek care (Gilson, 2003). Concrete levers include transparent and consistent public health communication, visible service improvements such as shorter waiting times and simplified appointment or referral procedures, continuity-of-care arrangements such as family-doctor contracting, and credible complaint-resolution mechanisms. These approaches may be more relevant to strengthening institutional trust than to promoting trust across all social relationships (Gilson, 2003; Putnam, 2000).

However, trust alone is insufficient to promote primary care gatekeeping. The absence of a significant association between trust profiles and primary care facility choice indicates that facility-level decisions are shaped by structural determinants. As Yip et al. (2019) noted in their assessment of China’s healthcare reform, inadequate primary care capacity and weak referral mechanisms remain central reasons why patients bypass grassroots facilities. Strengthening primary care thus requires investment in service capacity and institutional incentives beyond trust-building.

### 4.4. Strengths, Limitations, and Future Directions

This study has several methodological strengths. It used a three-wave longitudinal design enabling simultaneous examination of profile stability and change; a person-centered approach capturing nonlinear trust configurations that variable-centered methods would obscure; systematic comparison of three measurement specifications to support cross-wave interpretability; and sensitivity analysis confirming that findings did not depend on a single health-domain indicator.

Several limitations should be noted. First, attrition from the baseline eligible sample to the balanced panel was substantial, a common concern in longitudinal cohort studies that may affect generalizability (Gustavson et al., 2012). Attrition analyses showed that loss to follow-up was driven mainly by age and was essentially unrelated to the baseline trust indicators, and inverse-probability-weighted estimates were consistent with the main results; nevertheless, selection on unobserved characteristics cannot be ruled out, and official CFPS design weights applicable to a three-wave balanced panel were not available in our analytic files, limiting strict population-level generalizability. Second, because the CFPS administered care-seeking questions only to respondents reporting illness or physical discomfort during the preceding two weeks, the regression analyses focused on individuals with a recent health need; accordingly, the findings should be interpreted as pertaining to illness-related care-seeking decisions rather than to all forms of health service utilization. Third, the study is observational; the time-lagged design clarifies temporal ordering but cannot support causal inference. Fourth, data collection in 2020 and 2022 coincided with the COVID-19 pandemic; although the partial invariance model allowed government and doctor trust to vary freely across waves, some observed transitions may reflect period effects rather than individual-level trust evolution. Fifth, regression analyses used modal posterior profile assignments. Average posterior probabilities were high (0.865–0.929), and sensitivity analyses using posterior-probability weighting and pseudo-class multiple imputation reproduced the main findings, suggesting limited impact of classification uncertainty; in addition, trust was measured using four single-item indicators available in the CFPS, which may not fully capture the multidimensional nature of social trust. Fully integrated one-step or bias-adjusted three-step estimation within a multi-wave LTA framework remains a refinement for future work (Vermunt, 2010; Asparouhov & Muthén, 2014). Finally, although province fixed effects indicated that time-invariant regional factors do not explain the observed associations, formal multilevel models incorporating time-varying contextual indicators (e.g., regional healthcare expenditure) were beyond the scope of this study and merit future investigation.

Future research could incorporate post-pandemic waves to disentangle period effects from individual change, link survey data with administrative health records to validate self-reported care-seeking, and test whether community-level interventions improving service accessibility can promote trust development and behavioral change.

## 5. Conclusions

This study identified three longitudinally comparable social trust profiles among Chinese adults and demonstrated that the Selective Institutional Trust profile, characterized by high trust in government, doctors, and neighbors combined with very low stranger trust, was consistently associated with higher odds of subsequent illness-related care-seeking. This association was replicated across two independent two-year intervals, remained robust after covariate adjustment, and was strongest among individuals who maintained this profile over time. By contrast, Higher Generalized Trust showed no significant behavioral advantage, and no trust profile was associated with primary care facility choice. These findings indicate that the behavioral relevance of social trust for health-seeking is configuration-specific rather than monotonic, and highlight the value of person-centered longitudinal approaches for understanding how multidimensional trust structures are associated with health service utilization.

## Figures and Tables

**Table 1:** Baseline sample characteristics (standard deviation) (N = 12,216).

Variable	N (%) or M (SD)
Demographics	
Female	6174 (50.5%)
Male	6042 (49.5%)
Age (years)	44.9 (14.9)
Residence and health	
Urban residence	6329 (52.3%)
Self-rated health (1–5)	3.03 (1.18)
Chronic disease	1896 (15.5%)
Socioeconomic and enabling	
Education (years)	8.55 (4.76)
Married	10,081 (82.5%)
Health insurance	11,263 (92.2%)
Health service experience	
Satisfaction with access (1–5)	3.62 (0.81)
Satisfaction with quality (1–5)	3.48 (0.89)
Hospitalized past year	1375 (11.3%)
Illness and care-seeking	
Two-week illness 2018	3675 (30.1%)
Two-week illness 2020	3215 (26.4%)
Sought care 2020 (among ill)	2064 (64.2%)
Two-week illness 2022	3316 (27.2%)
Sought care 2022 (among ill)	2121 (64.0%)

**Table 2:** Trust indicator means at each time (standard deviation) (N = 12,216).

Indicator	2018 M (SD)	2020 M (SD)	2022 M (SD)
Neighbor trust	6.74 (2.03)	6.73 (2.05)	6.65 (2.11)
Stranger trust	2.33 (2.19)	2.39 (2.22)	2.65 (2.30)
Government trust	5.01 (2.61)	5.69 (2.51)	5.52 (2.57)
Doctor trust	6.67 (2.35)	7.08 (2.25)	6.91 (2.31)

**Table 3:** (**A**) The fit indices of all profile models at T1, T2 and T3. (**B**) LTA profile number comparison. (**C**) Measurement invariance model comparison. (**D**) Profile composition by wave (modal assignment).

(A)									
Time	Profile	LL	AIC	BIC	aBIC	Entropy	LMR	BLRT	Class Ratio (%)
T1	2	−107,245	214,516	214,613	214,571	0.627	<0.001	<0.001	36.0/64.0
	3	−105,992	212,020	212,154	212,097	0.719	<0.001	<0.001	30.8/33.0/36.2
	4	−105,399	210,843	211,014	210,941	0.714	<0.001	<0.001	26.2/28.6/13.3/31.9
	5	−103,796	207,648	207,855	207,766	0.894	<0.001	<0.001	19.2/24.7/26.5/2.6/27.1
T2	2	−106,483	212,993	213,089	213,048	0.649	<0.001	<0.001	33.9/66.1
	3	−105,384	210,804	210,937	210,880	0.772	<0.001	<0.001	30.0/29.5/40.5
	4	−104,766	209,578	209,749	209,676	0.743	<0.001	<0.001	39.0/8.2/16.9/35.9
	5	−102,930	205,916	206,123	206,034	0.904	<0.001	<0.001	24.3/18.1/27.8/2.8/27.1
T3	2	−107,760	215,547	215,643	215,602	0.658	<0.001	<0.001	31.7/68.3
	3	−106,673	213,382	213,515	213,458	0.740	<0.001	<0.001	29.2/27.7/43.2
	4	−106,009	212,064	212,234	212,161	0.813	<0.001	<0.001	26.6/44.5/26.4/2.4
	5	−104,092	208,239	208,447	208,358	0.915	<0.001	<0.001	16.0/32.6/26.1/21.8/3.5
**(B)**									
**Model**		**Parameters**		**AIC**		**BIC**		**aBIC**	**Entropy**
2-profile LTA		41		636,687		636,990		636,860	0.763
3-profile LTA		62		627,418		627,878		627,681	0.793
**(C)**									
**Specification**		**Parameters**		**AIC**		**BIC**		**DBIC**	**Entropy**
Free estimation		62		627,418		627,878		(ref)	0.793
Partial invariance		50		627,519		627,889		+11	0.792
Full invariance		38		628,099		628,380		+502	0.790
**(D)**									
**Profile**			**T1 N (%)**		**T2 N (%)**			**T3 N (%)**	
Low Trust			3788 (31.0%)		3837 (31.4%)			3700 (30.3%)	
Higher Generalized Trust			4343 (35.6%)		4596 (37.6%)			5179 (42.4%)	
Selective Institutional Trust			4085 (33.4%)		3783 (31.0%)			3337 (27.3%)	

**Table 4:** Latent transition probabilities of trust at T1, T2, and T3.

T1 → T2	Low Trust	Higher Generalized Trust	Selective Institutional Trust
Low Trust	0.777	0.126	0.097
Higher Generalized	0.119	0.733	0.148
Selective Institutional	0.093	0.207	0.701
**T2** → **T3**			
Low Trust	0.769	0.165	0.066
Higher Generalized	0.087	0.807	0.106
Selective Institutional	0.091	0.231	0.677

**Table 5:** (**A**) Time-lagged logistic regression: trust profiles predicting care-seeking (ref = Low Trust). (**B**) Trust profiles predicting primary care facility choice (ref = Low Trust). (**C**) Stayer–mover analysis: care-seeking by trajectory (ref = Stable Low Trust). (**D**) Predictors of profile transitions.

(A)							
Interval	Model	Selective Institutional OR [95% CI]		*p*	Higher Generalized OR [95% CI]		*p*
T1–T2	M1	1.35 [1.13, 1.62]		0.001	1.04 [0.87, 1.25]		0.648
	M2	1.30 [1.08, 1.57]		0.005	1.06 [0.89, 1.27]		0.516
	M3	1.30 [1.08, 1.56]		0.006	1.11 [0.92, 1.33]		0.275
T2–T3	M1	1.43 [1.19, 1.71]		<0.001	1.07 [0.90, 1.27]		0.443
	M2	1.36 [1.13, 1.63]		0.001	1.07 [0.89, 1.27]		0.483
	M3	1.35 [1.12, 1.63]		0.002	1.08 [0.91, 1.29]		0.383
**(B)**							
**Interval**	**Model**	**Selective Institutional OR [95% CI]**		***p***	**Higher Generalized OR [95% CI]**		***p***
T1–T2	M3	0.97 [0.78, 1.20]		0.782	0.98 [0.78, 1.22]		0.829
T2–T3	M3	1.00 [0.80, 1.24]		0.971	0.85 [0.68, 1.06]		0.144
**(C)**							
**Pathway**		**T1–T2 N**	**OR [95% CI]**	***p***	**T2–T3 N**	**OR [95% CI]**	***p***
Stable Low Trust (ref)		967	-	-	949	-	-
Stable Selective Institutional		790	1.47 [1.19, 1.80]	<0.001	731	1.48 [1.19, 1.83]	<0.001
Stable Higher Generalized		709	1.03 [0.84, 1.26]	0.754	871	1.07 [0.89, 1.30]	0.469
Selective Institutional to Low		85	1.86 [1.10, 3.14]	0.020	117	0.98 [0.66, 1.47]	0.924
Low to Selective Institutional		94	0.96 [0.62, 1.49]	0.856	69	1.26 [0.74, 2.16]	0.397
**(D)**							
**Transition**		**Predictor**		**OR [95% CI]**		***p***	
Low to Selective Institutional (N = 3788; 8.4%)							
		Satisfaction with access		1.41 [1.19, 1.68]		<0.001	
		Hospitalized past year		0.60 [0.39, 0.92]		0.020	
		Age		1.01 [1.00, 1.02]		0.006	
		Female (vs. male)		0.75 [0.59, 0.95]		0.017	
Higher Generalized to Low (N = 4343; 12.0%)							
		Satisfaction with access		0.81 [0.71, 0.94]		0.004	
		Age		1.01 [1.00, 1.02]		<0.001	
		Female (vs. male)		0.83 [0.69, 1.00]		0.049	
Selective Institutional to Low (N = 4085; 7.5%)							
		No predictor significant		-		-	

Note: OR = odds ratio; CI = confidence interval. Low Trust is the reference group for the trust profiles. M1 adjusts for age and sex. M2 adjusts for variables in M1 plus self-rated health, chronic disease, and urban residence. M3 adjusts for variables in M2 plus education, marital status, and health insurance.

## Data Availability

The data presented in this study are openly available on the CFPS website (https://www.isss.pku.edu.cn/cfps/, accessed on 10 March 2026). Processed data generated for this study are available from the corresponding author upon reasonable request.

## Appendix A

### Appendix A.1

**Table A1:** Estimated profile indicator means by wave (partial measurement invariance model).

Indicator	Wave	Low Trust (Profile 1)	Higher Generalized Trust (Profile 2)	Selective Institutional Trust (Profile 3)
Neighbor trust	2018	5.43	7.34	7.21
	2020	5.43	7.34	7.21
	2022	5.43	7.34	7.21
Stranger trust	2018	1.26	4.75	0.80
	2020	1.26	4.75	0.80
	2022	1.26	4.75	0.80
Government trust	2018	2.82	5.90	6.10
	2020	3.61	6.47	6.85
	2022	3.29	6.33	6.74
Doctor trust	2018	4.67	7.23	7.96
	2020	5.15	7.64	8.35
	2022	4.89	7.46	8.28

Note. N = 12,216. Neighbor and stranger trust means are constrained equal across waves (partial measurement invariance). Government and doctor trust means are freely estimated at each wave. All values are on a 0–10 scale.

### Appendix A.2. Hierarchical Logistic Regression Results Under the Freely Estimated Measurement Model

**Table A2:** (**1**) Saw a doctor when ill (among those reporting illness). (**2**) Primary care facility choice (among care-seekers).

(1)			
2018 Profile → 2020 Saw Doctor	Model 1 OR [95% CI]	Model 2 OR [95% CI]	Model 3 OR [95% CI]
Reference: Low Trust			
Selective Institutional	1.39 [1.16, 1.67]	1.35 [1.12, 1.62]	1.34 [1.11, 1.61]
Higher Generalized	1.06 [0.89, 1.26]	1.08 [0.91, 1.30]	1.13 [0.94, 1.36]
**2020 Profile** → **2022 Saw Doctor**			
Reference: Low Trust			
Selective Institutional	1.46 [1.21, 1.75]	1.38 [1.15, 1.67]	1.37 [1.14, 1.66]
Higher Generalized	1.08 [0.91, 1.28]	1.07 [0.90, 1.28]	1.09 [0.91, 1.30]
**(2)**			
**2018 Profile** → **2020 Primary Care**	**Model 1** **OR [95% CI]**	**Model 2** **OR [95% CI]**	**Model 3** **OR [95% CI]**
Reference: Low Trust			
Selective Institutional	1.01 [0.82, 1.25]	0.95 [0.77, 1.18]	0.95 [0.77, 1.18]
Higher Generalized	0.91 [0.73, 1.13]	0.91 [0.73, 1.14]	0.97 [0.77, 1.21]
**2020 Profile** → **2022 Primary Care**			
Reference: Low Trust			
Selective Institutional	1.12 [0.91, 1.38]	1.03 [0.83, 1.28]	1.03 [0.83, 1.28]
Higher Generalized	0.86 [0.69, 1.06]	0.85 [0.68, 1.06]	0.85 [0.68, 1.07]

Model 1: demographics (age, gender, education, marital status). Model 2: +need/residence (self-rated health, chronic disease, urban/rural). Model 3: +SES/enabling (income, insurance type). *p* < 0.01, *p* < 0.001. OR = odds ratio; CI = confidence interval. Reference category: Low-Trust profile.

### Appendix A.3. Sensitivity Analysis: LTA Results Excluding Doctor-Trust Indicator

**Table A3:** (**1**) Model fit comparison. (**2**) Profile proportions. (**3**) Latent transition probabilities. (**4**) Profile indicator means (no doctor trust model).

(1)					
Model	Parameters	LL	AIC	BIC	Entropy
Main (4 indicators)	50	−313,709.365	627,518.730	627,889.255	0.792
No doctor (3 indicators)	50	−235,269.558	470,639.115	471,009.640	0.799
**(2)**					
**Profile**		**2018**	**2020**	**2022**	
Low Trust (C1)		35.8%	34.4%	33.3%	
Higher Generalized (C2)		37.2%	39.0%	43.2%	
Selective Institutional (C3)		26.9%	26.6%	23.5%	
**(3)**					
**Interval**	**From\To**	**Low Trust**	**Higher Generalized Trust**	**Selective Institutional Trust**	
2018 → 2020	Low Trust	0.82	0.139	0.041	
	Higher Generalized	0.117	0.743	0.139	
	Selective Institutional	0.023	0.235	0.742	
2020 → 2022	Low Trust	0.821	0.157	0.021	
	Higher Generalized	0.106	0.795	0.099	
	Selective Institutional	0.035	0.256	0.709	
**(4)**					
**Indicator**	**Wave**	**Low Trust**	**Higher Generalized Trust**	**Selective Institutional Trust**	
Neighbor trust	2018	5.40	7.40	7.59	
	2020	5.36	7.29	7.68	
	2022	5.19	7.26	7.60	
Stranger trust	2018	1.04	4.70	0.78	
	2020	1.13	4.64	0.73	
	2022	1.18	4.84	0.71	
Government trust	2018	3.20	5.80	6.31	
	2020	3.92	6.37	6.98	
	2022	3.61	6.25	6.89	

Note. N = 12,216. The no-doctor model uses 3 indicators (neighbor trust, stranger trust, government trust) instead of 4. Profile proportions and transition patterns are highly consistent with the main 4-indicator model, supporting the robustness of the identified profile structure. Means are freely estimated at each wave (full invariance not imposed in this sensitivity model).

## Appendix B. Additional Analyses Addressing Attrition, Classification Uncertainty, and Contextual Confounding

**Table A4:** Baseline characteristics of retained versus lost respondents (N = 29,356).

Variable	Retained (*n* = 12,216)	Lost (*n* = 17,140)	SMD	*p*
Female, %	50.5	50.4	0.00	0.764
Age, years	44.9	49.1	−0.26	<0.001
Neighbor trust (0–10)	6.74	6.73	0.00	0.988
Stranger trust (0–10)	2.33	2.25	0.04	0.002
Government trust (0–10)	5.01	5.16	−0.06	<0.001
Doctor trust (0–10)	6.67	6.75	−0.03	0.007
Two-week illness, %	30.1	32.7	−0.06	<0.001
Sought care if ill, %	75.0	77.5	−0.06	0.006
Primary care if sought, %	59.1	58.1	0.02	0.089
Hospitalized past year, %	11.3	14.5	−0.10	<0.001
Total medical cost, yuan	2690	4074	−0.10	<0.001
Out-of-pocket cost, yuan	2581	3595	−0.10	<0.001
Satisfaction with access (1–5)	3.62	3.64	−0.02	0.086
Satisfaction with quality (1–5)	3.48	3.52	−0.04	<0.001

Note. SMD = standardized mean difference. Retained = respondents in the balanced three-wave panel; Lost = baseline-eligible respondents not retained across all three waves.

**Table A5:** Attrition sensitivity analysis of fully adjusted care-seeking models: unweighted versus inverse-probability-weighted estimates (ref = Low Trust).

Interval	Model	Selective OR [95% CI]	*p*	Higher Generalized OR [95% CI]	*p*
2018 → 2020	Unweighted	1.30 [1.08, 1.56]	0.006	1.11 [0.92, 1.33]	0.275
2018 → 2020	IPW	1.29 [1.07, 1.55]	0.007	1.10 [0.92, 1.33]	0.294
2020 → 2022	Unweighted	1.35 [1.12, 1.63]	0.002	1.08 [0.91, 1.29]	0.383
2020 → 2022	IPW	1.33 [1.10, 1.60]	0.003	1.09 [0.91, 1.30]	0.365

Note. Unweighted and IPW estimates are based on the fully adjusted model (M3 in Table 5), adjusted for age, sex, self-rated health, chronic disease, urban residence, education, marital status, and health insurance Stabilized inverse-probability-of-retention weights had a mean of 1.00 (range 0.73–4.52; 1st and 99th percentiles, 0.78 and 1.42) and were truncated at the 1st and 99th percentiles; the overall three-wave panel retention rate was 41.6%. OR = odds ratio; CI = confidence interval.

**Table A6:** Average posterior probabilities for the assigned profile, by wave.

Wave	Low Trust	Selective Institutional	Higher Generalized
2018	0.878	0.866	0.928
2020	0.895	0.890	0.919
2022	0.883	0.865	0.929

Note. Average posterior probability for the modally assigned profile. All off-diagonal average posterior probabilities were ≤0.088.

**Table A7:** Covariate-adjusted average predicted probabilities of care-seeking when ill.

Interval	Profile	Predicted Probability [95% CI]	Difference vs. Low
2018 → 2020	Low Trust	62.0% [59.3, 64.7]	-
2018 → 2020	Selective Institutional	67.6% [64.8, 70.4]	+5.6 pp
2018 → 2020	Higher Generalized	64.2% [61.1, 66.9]	+2.2 pp
2020 → 2022	Low Trust	61.6% [58.8, 64.2]	-
2020 → 2022	Selective Institutional	68.0% [64.8, 70.8]	+6.5 pp
2020 → 2022	Higher Generalized	63.3% [60.4, 66.0]	+1.7 pp

**Table A8:** Classification-uncertainty sensitivity analysis of fully adjusted care-seeking models: posterior-probability weighting and pseudo-class multiple imputation (ref = Low Trust).

Method	Interval	Selective OR [95% CI]	*p*	Higher Generalized OR [95% CI]	*p*
Posterior-probability weighting	2018 → 2020	1.31 [1.16, 1.47]	<0.001	1.16 [1.03, 1.30]	0.018
Posterior-probability weighting	2020 → 2022	1.37 [1.21, 1.55]	<0.001	1.10 [0.98, 1.25]	0.104
Pseudo-class MI (200 draws)	2018 → 2020	1.31 [1.06, 1.62]	0.014	1.15 [0.94, 1.41]	0.161
Pseudo-class MI (200 draws)	2020 → 2022	1.37 [1.11, 1.69]	0.003	1.10 [0.91, 1.34]	0.314

**Table A9:** Fully adjusted models with province fixed effects (ref = Low Trust).

Interval	Specification	Selective OR [95% CI]	*p*	Higher Generalized OR [95% CI]	*p*
2018 → 2020	M3	1.30 [1.08, 1.56]	0.006	1.11 [0.92, 1.33]	0.275
2018 → 2020	M3 + province FE	1.25 [1.04, 1.51]	0.020	1.07 [0.89, 1.29]	0.473
2020 → 2022	M3	1.35 [1.12, 1.63]	0.002	1.08 [0.91, 1.29]	0.383
2020 → 2022	M3 + province FE	1.34 [1.11, 1.62]	0.003	1.05 [0.88, 1.26]	0.564

Note. M3 = fully adjusted model (age, sex, self-rated health, chronic disease, urban residence, education, marital status, health insurance). Province fixed effects absorb all time-invariant province-level differences. OR = odds ratio; CI = confidence interval.
